# Iodoform as a Dressing to Fresh Wounds

**Published:** 1882-08-20

**Authors:** Lindsay Johnson

**Affiliations:** Ga.


### IODOFORM AS A DRESSING TO FRESH WOUNDS.

By LINDSAY JOHNSON, M. D., OF GA.

Last February I was called to see a young man at Bartow Iron Works—a station on the W. & A. R. R.—who had, the evening before, been run over by a freight train, his right ankle being so badly mangled that amputation of the lower third of the leg was at once performed. At the time of the operation no antiseptic precaution whatever was taken. Patient was very much exhausted from loss of blood, long suffering (eighteen hours having elapsed since reception of injury) and shock. Wound was simply dressed with cold cloths, and directions left with the nurse to keep dressing wet till my return next day. Primary dressing was then removed and iodoform well sprinkled over surface of wound, the stump enveloped by a wide piece of smoothe cloth, greased with vaseline to prevent sticking, and over the whole a roller bandage, to be unmolested for four days. At the expiration of this time I returned, and upon removing the dressing found wound perfectly sweet, presenting a healthy, granulating surface with slight sero-sanguineous flow. Sutures were all removed, wound cleansed and dressed in same way. Directions left with nurse to continue the use of iodoform, removing dressing and reapplying once in twenty-four hours.

After this I saw patient every four days. No pain or muscular twitching in attendance; slept well without the aid of an anodyne or hypnotic; appetite good throughout; suppuration slight, and from first to last not the faintest odor ever perceptible. In two weeks from date of accident patient was up and on crutches; no untoward symptom having shown itself during treatment, and nothing used as a dressing save the iodoform.

Again: Virgil Cox, æt 22 years, an employee of the W. & A. R. R., while engaged in coupling cars at Cartersville, Ga., on the night of April 8th, had his clothing caught in a brake and was thrown beneath the cars, one wheel passing obliquely over right leg between the knee and ankle. The cars being in motion he was carried the distance of forty feet, receiving in his passage over the track-railing and ends of cross-ties, deep lacerations on either side of the perineum, besides numerous superficial abrasions and contusions. When I first saw him it seemed almost a forlorn hope to attempt surgical interference; yet not feeling satisfied to stand passively by and make no effort at saving a valuable life, had him taken from the office floor of a hotel, where he had been borne by friends, and placed upon a table. Ably assisted by Drs. W. W. and R. W. Leake, I amputated thigh at the lower third, about 12 o'clock—two hours after reception of the injury. With all the unfavorable circumstances surrounding such an operation at such an hour—the profound shock, excessive exhaustion from previous loss of blood, it appeared almost impossible that patient should survive even a few hours. Stump and other wounds all dressed, our full energies were employed in reviving patient from chloroform stupor, and establishing general reaction. Suffice it to say, he at length "came around." For the first few days carbolic acid in conjunction with iodoform was used as a dressing and general disinfectants.

About the fifth day sutures were removed; wounds, stump and perineum presenting fresh and healthy granulating surfaces. After this iodoform alone was used, previous experience having thoroughly convinced me that as a local anæsthetic and antiseptic, and as a remedy against painful muscular jerking, this agent was par excellence. At each dressing the wounds were well cleansed of the sero-sanguineous matter which invariably follows amputations and wounds of like character, and a fresh supply of iodoform sprinkled over the surfaces, covered with several layers of soft cloth, and the whole length of stump enveloped in a roller bandage. This method was persisted in during the whole course of treatment; and, as in the case previously mentioned, not the slightest odor, at any time, could be detected, nor was pain present in a degree demanding the employment of an anodyne. The perineal wounds, which gave us most concern, healed with great kindness and rapidity. Indeed, I have yet to see a case that, all in all, resulted so satisfactorily. Notwithstanding the intervention of acute rheumatism, the young man was able to take crutches and walk the distance of seventy yards, get upon the cars and go to his home, about forty miles distant, the 48th day from the time of injury. His wounds all healed, and in good general health and spirits.

Now, in taking a retrospect of the action of iodoform in this last mentioned case, when the patient was subjected to powerful shock, numerous abrasions and contusions, deep lacerations in and near vital organs, and an exhaustive operation, I am forced to the conclusion that most of what has been said of the intoxicating and poisonous effect of this agent is all "*bosh.*" Not for a single moment could elevation of temperature, headache, depression, loss of appetite, or other untoward symptom be ascribed to this remedy. That some individuals may exhibit an idiosyncrasy in respect to iodoform, I have no doubt; while in a great majority of cases I am led to believe nothing of a toxic nature could be imputed to its employment. As in opium, ipecac and various other drugs, if unfavorable constitutional peculiarities should arise, the discontinuance of its use would at once, I am sure, put a stop to the trouble.

I will only add, that in a recent case of deep knife wound—penetrating the thoracic cavity from behind—I used iodoform alone as a dressing, receiving from its use the full benefit of a safe antiseptic, a reliable local anæsthetic and rapid promoter of healthy granulation. Am now treating a serious gun-shot wound with much satisfaction, with this agent alone.

In a great number of other cases, differing in character, yet all fresh wounds, I have employed the iodoform to my full and entire satisfacion.

